# The association between diet quality index-international and dietary diversity score with preeclampsia: a case–control study

**DOI:** 10.1186/s12905-024-03023-0

**Published:** 2024-03-21

**Authors:** Parastoo Tolou Hayat, Bahram Pourghassem Gargari, Parvin Sarbakhsh

**Affiliations:** 1 Student Research Committee, Department of Biochemistry and Diet Therapy, Faculty of Nutrition and Food Sciences, Tabriz University of Medical Sciences, Tabriz, Iran; 2 Nutrition Research Centre, Department of Biochemistry and Diet Therapy, Faculty of Nutrition and Food Sciences, Tabriz University of Medical Sciences, Tabriz, Tell Iran; 3 Department of Statistics and Epidemiology, School of Public Health, Tabriz University of Medical Sciences, Tabriz, Iran

**Keywords:** Dietary quality index, Dietary diversity score, Preeclampsia, Nutritional assessment, Hypertension

## Abstract

**Background:**

Preeclampsia is a significant complication that occurs during the second half of pregnancy. Recent studies have indicated that dietary factors play a crucial role in the development of preeclampsia. The Diet Quality Index-International (DQI-I) and Dietary Diversity Score (DDS) are appropriate indices for assessing the quality of foods, meals, and diets. This study aimed to investigate the relationship between DQI-I, DDS, and preeclampsia.

**Methods:**

This study utilized a case-control design. A total of 90 newly diagnosed preeclampsia cases and 90 healthy controls were included from a referral hospital in Tabriz, Iran. DQI-I and DDS were calculated based on information obtained from a reliable Food Frequency Questionnaire consisting of 168 food items, which assessed participants’ usual diet. Logistic regression analysis adjusted for age, body mass index, education, family history of preeclampsia, and total energy intake was used to estimate odds ratios (ORs).

**Results:**

The mean age and pre-pregnancy body mass index of the participants were: 27.14 ± 4.40 years and 26.09 ± 3.33 kg/m^2^, respectively. After adjusting for various confounders, we found significant inverse association between the risk of developing preeclampsia and both DQI-I and DDS. The highest quartile of DQI-I had a significantly lower risk of developing preeclampsia compared to the first quartile (OR = 0.02, 95% CI [0.005, 0.08]) (*P* < 0.001). Similarly, the highest quartile of DDS had a significantly lower risk of developing preeclampsia compared to the first quartile (OR = 0.09, 95% CI [0.03, 0.31]) (*P* = 0.001).

**Conclusions:**

Our findings suggest that maintaining a high-quality and diverse diet is associated with a lower risk of preeclampsia. Further studies are needed to confirm these associations and explore potential causal relationships.

## Introduction

Preeclampsia is characterized by hypertension that occurs after 20 weeks of gestational age, along with proteinuria or other signs of organ damage. It is a significant cause of both maternal and perinatal morbidity and mortality, particularly when it occurs early in pregnancy [1]. Hypertensive disorders of pregnancy affect around 10% of pregnancies worldwide, with preeclampsia accounting for 3–5% of cases [2]. According to the World Health Organization (WHO), the incidence of preeclampsia is seven times higher in developing countries compared to developed counties with rates of 2.8% versus 0.4% of live births, respectively [3].

Preeclampsia induces a poly-systemic syndrome characterized by vasoconstriction, metabolic shifts, endothelial dysfunction, heightened inflammatory response, and reduced organ perfusion. Offspring born to mothers with preeclampsia face an elevated risk of various disorders, including endocrine, metabolic, and nutritional issues during puberty [4]. A meta-analysis by Bartsch et al. identified antiphospholipid syndrome, prior preeclampsia, pre-gestational diabetes, chronic hypertension, assisted reproductive technology, and high BMI as strongly associated risk factors for preeclampsia [5]. Despite a comprehensive understanding of clinical presentation, diagnostic criteria, and management, the underlying cause of preeclampsia remains poorly understood. A widely accepted hypothesis points to an abnormal placenta leading to significant maternal physiological dysfunction. This abnormal placenta is thought to give rise to malformed spiral arteries, placental ischemia, hypoxia, and oxidative stress, contributing to the etiology of preeclampsia [6, 7].

Contrastingly, observational studies have indicated associations between the incidence of preeclampsia and various dietary components, including macronutrients, micronutrients, dietary fiber, alcohol, caffeine, individual foods, and overall dietary patterns [8–10]. However, trials focused on preventing preeclampsia have produced diverse and sometimes conflicting results across different studies [11–13].

Dietary Quality Indices (DQIs) are designed to evaluate the overall quality of a person’s diet and categorize individuals based on the healthiness of their eating behavior [14]. The DQI-I developed by Kim et al. [15], assesses four aspects of a healthy diet: moderation, balance, variety, and adequacy. The total DQI-I score ranges from 0 to 100, with a higher score indicating a higher quality diet [16]. The Diet Quality Index-International (DQI-I) is directly linked to the risk of noncommunicable chronic diseases (NCCDs). A poor score on the DQI-I has been shown to increase the risk of obesity and its associated conditions such as diabetes, cancer, and cardiovascular diseases [17–21]. On the other hand, the Dietary Diversity Score (DDS) measures the number of different food items or groups consumed over a specific period, either at the household or individual level [22]. This concept is widely recognized as an important aspect of dietary quality. The idea is that consuming a variety of foods ensures the intake of essential nutrients, leading to improved diet quality and better health outcomes [23]. Cosuming a diverse range of foods from different groups is associated with higher likelihood of meeting nutrient requirements [24], higher levels of antioxidant blood markers [25], and lower risk factors for cardiovascular diseases [26].

The role of diet in preeclampsia has been the subject of investigation for years, but hypotheses have varied, resulting in conflicting results in studies. Notably, there is a lack of research evaluating the association between dietary diversity and the dietary quality index with preeclampsia in the Middle East. This region has unique dietary patterns, characterized by significant consumption of refined carbohydrates, especially rice and bread. To fill this gap, we conducted a case-control study to explore the relationship between dietary diversity, dietary quality, and the risk of preeclampsia in this specific region.

## Methods

### Participants

A case–control study was conducted focusing on individuals diagnosed with preeclampsia. The sample size was determined based on a previous study [4] and by using G power software [27]. The sample size formula for independent groups, considering group matching and dependency, a 0.15 Pearson’s correlation coefficient, 96% power, and 95% confidence was applied. Each group required 87 participants, which was increased to 90 to account for potential sample drops. Therefore, this study included 90 preeclampsia cases and 90 healthy pregnant controls aged 20 to 35 years, from Tabriz referral hospital, Iran. The diagnosis of preeclampsia was confirmed by examining medical records and considering criteria such as high blood pressure (≥ 140mmHg systolic or ≥ 90mmHg diastolic on two occasions with an interval of 6 h), excretion of more than 0.3 g of protein in a 24-hour urine sample, and gestational age of more than 20 weeks. The control group consisted of healthy women from the same hospital who were age-matched with the cases and were at least 20 weeks pregnant. Exclusion criteria for both cases and controls included a history of chronic illnesses, malignancies, autoimmune diseases, and other inflammatory or infectious disorders. Face-to-face interviews were conducted to gather information on demographics, medical history, medications, diet, pregnancy, education, and family history of preeclampsia. Weight and height were assessed before- and during pregnancy to calculate pre- and during pregnancy body mass index (BMI). We also calculated gestational weight gain (GWG) for both groups by subtracting pre-pregnancy weight from weight during pregnancy. Informed consent was obtained from each participant prior to enrolment. The study protocol was approved by the ethics committee of Tabriz University of Medical Science (Ethic’s Approval code: IR.TBZMED.REC.1401.523).

### Dietary assessment

Dietary intake was assessed using a validated 168-item semi-quantitative food frequency questionnaire (FFQ). This questionnaire was designed based on the Willett method and modified for Iranian foods. It estimates the frequency of consuming each item over the past year. Its validity and reliability has been confirmed for Iranian populations [28]. An experienced dietitian conducted face-to-face interviews to complete FFQs for all participants. The mean daily intake of each food was estimated by converting usual portion sizes to grams using household measures. The total energy, macronutrient, and micronutrient intake were calculated using a modified version of Nutritionist IV software tailored for Iranian foods.

### Diet quality index-international assessment (DQI-I)

Diet quality was evaluated using the DQI-I, which consists of four main components. The first component is food variety, which measures the overall variety of foods across different food groups and within-group variety of protein sources. This component is scored on a scale of 0 to 20 points. The second component is adequacy, which evaluates the amounts of vegetables, fruits, grains, fiber, protein, iron, calcium, and vitamin C in the diet. This component is scored on a scale of 0 to 40 points. The third component is moderation, which takes into account the intake of total fat, saturated fat, cholesterol, sodium, and empty calorie foods. This component is scored on a scale of 0 to 30 points. The fourth component is overall balance, which considers the ratio of macronutrients and fatty acids in the diet. This component is scored on a scale of 0 to 10 points. The total DQI-I score, is calculated by combining these four components and ranges from 0 to 100, with 0 indicates the poorest dietary quality, while a score of 100 indicates the highest dietary quality [16].

### Dietary diversity score assessment (DDS)

The dietary diversity score was assessed using the method outlined by Kant et al. [29]. This approach considered five groups: grains, vegetables, fruits, meats, and dairy, which align with the USDA food guide pyramid. The grains group included refined bread, macaroni, whole grain bread, corn flakes, biscuits, refined flour, and rice. Fruit was defined as a combination of fruit and fruit juice, berries, and citrus fruits. Vegetables encompassed potato, tomato, other starchy vegetables, legumes, yellow vegetables, green vegetables, and other vegetables. The meat group comprised red meat, poultry, fish, and eggs, while the dairy group included milk, yogurt, and cheese.

### Statistical analysis

To compare classified variables between groups, either the Chi-square test or Fisher’s exact test was employed. Prior to selecting the statistical test, the normality of data distribution for each variable was assessed using the Kolmogorov-Smirnov test. Subsequently, the independent samples t-test or Mann-Whitney U test was used to compare continuous variables with normal and abnormal distributions between groups. Age-adjusted and multivariate logistic regression models were applied to estimate odds ratios (ORs) and 95% confidence intervals (CIs) for DQI-I and DDS in relation to the risk of preeclampsia. The crude model did not include any adjustments, whereas the second model, accounted for the effect of energy. The third model, included additional adjustments for age, body mass index, education, family history of preeclampsia, and total energy intake. The statistical analysis of the study data was conducted using SPSS software (IBM SPSS Statistics 26), and a *P*-value < 0.05 was considered as the threshold for significance at all stages.

## Results

In this study, 90 participants were pregnant women diagnosed with preeclampsia, while the remaining 90 individuals were controls with a healthy pregnancy of more than 20 weeks. The demographic questionnaire results showed that the pregnancy was mostly unplanned, and the participants were both primiparous and multiparous. All participants received the standard pregnancy supplements recommended by the Iranian Ministry of Health and Medical Education, including 150 mg of ferrous sulfate, 400mcg of folate, and a multivitamin/mineral supplement.

The average age and height of cases were 27.51 ± 4.27(y) and 1.64 ± 0.06 (m), respectively. The average age and height of controls were 26.77 ± 4.52(y) and 1.66 ± 0.06 (m), respectively. Pre-pregnancy weight among cases and controls was: 70.67 ± 10.74 (kg) and 70.98 ± 10.65, respectively. The pre-pregnancy BMI among the cases and controls was 26.35 ± 3.35(kg/m^2^) and 25.84 ± 3.31(kg/m^2^), respectively. The difference was not significant. Preeclampsia subjects had a higher during pregnancy BMI than healthy non-preeclampsia subjects (30.31 ± 2.77(kg/m^2^) and 27.95 ± 2.26(kg/m^2^), respectively, *P* < 0.001). Gestational weight gain was higher in women with preeclampsia than in healthy women (10.47 ± 5.35 kg vs. 5.66 ± 7.46 kg, *P* < 0.001).

Cases had a higher family history of high blood pressure during pregnancy compared to the controls (*P* = 0.004). Table 1 shows the distribution of dietary intakes of macronutrients and micronutrients among cases and controls. The case group had a higher intake of total fat (111.66 ± 31.70 g/d vs. 93.42 ± 25.61 g/d), and monounsaturated fatty acid (MUFA, 37.93 ± 11.34 g/d vs. 31.50 ± 8.63 g/d) compared to the control group. There were no significant differences between the groups in terms of total energy intake, protein, carbohydrate, PUFA, calcium, sodium, whole grain, refined grain, white meat, red meat, fish, salt, nuts, and dairy products intake. The general characteristics of the participants across quartiles of DQI-I and DDS are shown in Table 2. No significant differences were found in terms of general characteristics across quartiles of DQI-I and DDS. The dietary intakes of cases and controls across quartiles of DQI-I and DDS are presented in Tables 3 and 4. Those in the top quartile of DQI-I had higher intakes of fruit, vegetables, dairy products, legumes, refined grains, energy, protein, carbohydrates, SFA, calcium, sodium and fiber compared to individuals in the bottom quartile. No other significant differences were found in terms of dietary intakes across quartiles of DQI-I. Those in the top quartile of DDS had higher intakes of fruit, vegetables, white meat, red meat, fish, dairy products, legumes, nuts, energy, protein, fat, carbohydrates, SFA, MUFA, calcium, sodium and fiber compared to individuals in the bottom quartile. No other significant differences were found in terms of dietary intakes across quartiles of DQI-I and DDS. The multivariable-adjusted OR and 95% CI for preeclampsia across quartiles of DQI-I and DDS are shown in Table 5. After controlling for energy intake, an inverse and significant association was found between DQI-I and DDS and the risk of preeclampsia (OR 0.02, 95% CI 0.008, 0.09) and (OR 0.10, 95% CI 0.03,0.33), respectively. This finding was also observed when further adjustments were made for age, education, BMI, and family history. Participants in the highest quartile of DQI-I and DDS were 98% and 91% less likely to have preeclampsia compared to those in the lowest quartile (OR 0.02, 95% CI 0.005, 0.08) and (OR 0.09, 95% CI 0.03,0.31), respectively.

**Table 1:** Distribution of dietary intakes of macro- and micronutrients among cases and controls

Variables	Cases ^a^ (*n* = 90)	Controls (*n* = 90)	*P* value
Total energy intake (kcal/day)	2763.81 ± 618.32	2616.13 ± 625.63	0.11
Protein (g/day)	91.94 ± 29.06	92.72 ± 27.52	0.85
Carbohydrate (g/day)	358.80 ± 88.08	364.58 ± 88.24	0.66
Total fat (g/day)	111.66 ± 31.70	93.42 ± 25.61	**< 0.001**
MUFA (g/day)	37.93 ± 11.34	31.50 ± 8.63	**< 0.001**
PUFA (g/day)	25.16 ± 8.09	23.06 ± 7.69	0.07
Calcium (mg/day)	1115.76 ± 369.23	1184.82 ± 345.16	0.19
Na (mg/day)	6205.48 ± 1799.65	6140.28 ± 1546.41	0.07
K (mg/day)	3582.33 ± 971.35	3748.00 ± 1019.76	**< 0.001**
Mg (mg/day)	398.05 ± 103.26	425.00 ± 110.50	**< 0.001**
Zinc (mg/day)	12.97 ± 4.68	12.88 ± 3.93	**< 0.001**
Fiber ( g/day)	52.53 ± 23.70	62.61 ± 26.53	**0.008**
Whole Grain (g/day)	72.72 ± 53.83	87.55 ± 66.30	0.10
Refined Grain (g/day)	370.56 ± 133.02	379.17 ± 127.62	0.65
White Meat (g/day)	39.27 ± 34.02	45.11 ± 33.16	0.24
Red Meat (g/day)	43.57 ± 56.68	30.49 ± 31.48	0.57
Fish (g/day)	7.52 ± 11.99	8.43 ± 16.75	0.67
Salt (g/day)	8.23 ± 3.28	7.85 ± 2.44	0.37
Fruits (g/day)	298.26 ± 163.31	379.23 ± 179.09	**0.002**
Vegetables (g/day)	262.79 ± 137.28	313.19 ± 135.52	**0.01**
Legumes (g/day)	21.65 ± 14.63	26.98 ± 13.22	**0.01**
Nuts (g/day)	16.70 ± 26.79	18.89 ± 23.31	0.55
Dairy Products (g/day)	324.16 ± 231.53	323.85 ± 180.61	0.99
DDS	4.63 ± 1.66	5.44 ± 1.70	**0.002**
DQI-I	52.64 ± 7.94	60.46 ± 8.55	**< 0.001**

^a^Pregnant women with preeclampsia disease and a gestational age of more than 20 weeks.

SFA: Saturated fatty acids, MUFA: Monounsaturated fatty acids, PUFA: Polyunsaturated fatty acids, SD: Standard deviation, DDS: Dietary diversity score, DQI-I: Dietary quality index-international.

Values are mean ± SD or median (25th, 75th percentiles) for continuous.

Independent samples t-test was used.

Significant values are shown in bold.

**Table 2:** General characteristics of study participants across quartiles (Q) of dietary quality index-international (DQI-I) and dietary diversity score (DDS) (Mean values and standard deviations; percentages)

Quartiles of DQI-I and DDS										
	DQI-I					DDS				
	Q1	Q2	Q3	Q4	*P* value ^a^	Q1	Q2	Q3	Q4	*P* value ^a^
Age( years)	27.48 ± 3.70	26.52 ± 4.35	27.25 ± 4.67	27.33 ± 4.88	0.73	26.95 ± 4.01	27.53 ± 4.54	26.73 ± 4.38	27.44 ± 4.66	0.78
Pre-pregnancy BMI(kg/m^2^) ^b^	25.78 ± 3.11	26.67 ± 3.36	25.49 ± 3.08	26.41 ± 3.71	0.30	25.42 ± 3.09	26.03 ± 2.74	26.60 ± 3.97	26.63 ± 3.26	0.27
Education					0.10					0.88
Illiterate	4(9)	6(13)	4(9)	4(9)		5(12)	4(9)	3(7)	6(13)	
Primary	4(9)	6(13)	8(18)	11(24)		8(19)	6(14)	8(18)	5(11)	
Secondary and high school	14(31)	21(46)	19(43)	22(49)		16(37)	18(42)	18(40)	23(51)	
University	23(51)	13(28)	13(29)	8(18)		14(33)	15(35)	16(36)	11(24)	
Family history ^c^					0.40					0.87
Yes	12(27)	10(22)	6(14)	12(27)		10(23)	10(23)	8(18)	11(24)	
No	33(73)	36(78)	38(86)	33(73)		33(77)	33(77)	37(82)	34(76)	

^a^ significant at p˂0.05.

^b^ Body mass index.

^c^ Family history: a record of the diseases and health conditions within your family.

Data was obtained from ANOVA or χ2 tests, as appropriate.

**Table 3:** Dietary and nutrient intakes of study participants across quartiles (Q) of the dietary quality index-international (DQI-I)

Quartiles of DQI-I					
	Q1	Q2	Q3	Q4	*P* value
Food groups (g/d)					
Fruits	244.60 ± 132.33	292.79 ± 114.48	354.78 ± 183.95	464.20 ± 184.15	**< 0.001**
Vegetables	226.97 ± 114.71	264.14 ± 134.92	293.49 ± 143.25	368.01 ± 122.71	**< 0.001**
White meat	36.10 ± 25.74	44.11 ± 40.55	38.56 ± 34.04	49.87 ± 31.76	0.21
Red meat	31.65 ± 40.98	41.45 ± 46.14	34.69 ± 42.93	40.19 ± 54.35	0.71
Fish	4.36 ± 7.24	8.75 ± 14.03	9.48 ± 21.86	9.33 ± 10.96	0.28
Dairy products	261.95 ± 203.00	378.40 ± 276.42	300.58 ± 138.14	353.35 ± 167.19	**0.03**
Legumes	17.50 ± 10.94	24.40 ± 13.22	27.99 ± 14.48	27.46 ± 15.54	**0.01**
Nuts	10.77 ± 12.24	18.17 ± 26.60	18.79 ± 19.97	23.47 ± 34.76	0.11
Whole grain	62.41 ± 50.40	84.79 ± 62.79	85.20 ± 64.27	88.15 ± 62.70	0.15
Refined grain	293.25 ± 98.34	354.56 ± 123.69	417.77 ± 122.29	435.28 ± 126.73	**< 0.001**
Salt	8.04 ± 2.72	8.51 ± 2.89	7.97 ± 3.22	7.61 ± 2.72	0.53
SSB	70.55 ± 47.00	84.83 ± 79.83	87.51 ± 62.12	75.46 ± 65.50	0.57
Nutrients					
Energy (kcal/d)	2259.00 ± 514.16	2771.00 ± 536.87	2793.15 ± 561.85	2937.22 ± 670.62	**< 0.001**
Protein (g/d)	74.09 ± 22.45	96.03 ± 26.00	94.85 ± 28.73	104.34 ± 27.05	**< 0.001**
Fat (g/d)	96.84 ± 27.83	111.48 ± 33.39	102.01 ± 27.37	99.63 ± 30.44	0.10
Carbohydrate (g/d)	281.71 ± 63.82	356.70 ± 63.29	387.52 ± 66.38	421.51 ± 90.03	**< 0.001**
SFA (g/d)	27.68 ± 10.50	33.47 ± 11.72	28.80 ± 8.80	28.24 ± 9.60	**0.02**
PUFA (g/d)	23.01 ± 7.74	25.24 ± 8.04	24.68 ± 7.55	23.50 ± 8.46	0.51
MUFA (g/d)	34.15 ± 10.55	37.46 ± 12.08	34.04 ± 9.84	33.14 ± 9.29	0.22
Ca (g/d)	885.14 ± 303.74	1247.58 ± 378.26	1150.09 ± 233.69	1316.18 ± 345.99	**< 0.001**
Na (mg/d)	5553.53 ± 1451.45	6306.86 ± 1732.43	6308.87 ± 1666.59	6522.31 ± 1716.81	**0.03**
Dietary fiber (g/d)	39.64 ± 18.74	55.50 ± 21.20	60.70 ± 24.02	74.54 ± 25.52	**< 0.001**

DQI-I: Dietary Quality Index-International, SSB: Sugar-sweetened beverages, SFA: Saturated fatty acids, PUFA: Polyunsaturated fatty acids, MUFA: Monounsaturated fatty acids, Ca: Calcium, Na: Sodium

The values are presented as mean ± SD.

Using one-way ANOVA.

Significant values are shown in bold.

**Table 4:** Dietary and nutrient intakes of study participants across quartiles (Q) of dietary diversity score (DDS)

Quartiles of DDS					
	Q1	Q2	Q3	Q4	*P* value
Food groups (g/d)					
Fruits	225.89 ± 123.06	295.13 ± 137.25	351.47 ± 135.45	487.59 ± 188.54	**< 0.001**
Vegetables	224.60 ± 119.06	254.92 ± 131.81	280.45 ± 110.33	385.44 ± 134.56	**< 0.001**
White meat	30.64 ± 26.36	42.66 ± 36.73	40.46 ± 27.17	56.78 ± 38.49	**0.003**
Red meat	19.91 ± 16.38	36.33 ± 40.65	41.60 ± 41.41	51.47 ± 67.46	**0.01**
Fish	3.69 ± 7.37	3.37 ± 5.66	9.97 ± 14.41	14.73 ± 21.84	**< 0.001**
Dairy products	224.99 ± 191.73	274.65 ± 198.56	368.49 ± 172.08	426.60 ± 214.95	**< 0.001**
Legumes	19.24 ± 10.98	20.34 ± 13.28	25.72 ± 13.26	30.99 ± 15.84	**< 0.001**
Nuts	9.00 ± 9.85	17.17 ± 16.67	22.61 ± 30.28	22.36 ± 33.69	**0.03**
Whole grains	64.84 ± 59.87	83.91 ± 56.26	73.66 ± 46.17	94.72 ± 72.22	0.10
Refined grains	349.76 ± 152.37	377.36 ± 119.96	383.09 ± 107.01	389.49 ± 141.46	0.51
Salt	8.10 ± 2.78	7.69 ± 3.18	8.32 ± 3.08	8.06 ± 2.48	0.78
SSB	66.17 ± 50.00	75.63 ± 54.73	91.67 ± 77.51	87.37 ± 71.56	0.24
Nutrients					
Energy (kcal/d)	2246 ± 35	2632.00 ± 436.10	2801.54 ± 54	3108.50 ± 685.13	**< 0.001**
Protein (g/d)	69.13 ± 18.74	89.20 ± 19.49	96.86 ± 18.94	114.90 ± 32.54	**< 0.001**
Fat (g/d)	23.81 ± 8.81	28.23 ± 8.37	32.13 ± 9.32	34.75 ± 11.58	**< 0.001**
Carbohydrate (g/d)	300.31 ± 79.18	353.47 ± 63.83	374.91 ± 67.73	419.26 ± 96.62	**< 0.001**
SFA (g/d)	23.81 ± 8.81	28.23 ± 8.37	32.13 ± 9.32	34.75 ± 11.58	**< 0.001**
PUFA (g/d)	23.17 ± 7.23	24.53 ± 8.19	24.45 ± 7.79	24.87 ± 8.69	0.76
MUFA (g/d)	30.69 ± 9.21	34.83 ± 10.33	36.33 ± 10.66	37.86 ± 10.71	**0.009**
Ca (g/d)	900.03 ± 339.95	1060.62 ± 256.47	1230.16 ± 287.14	1417.77 ± 332.94	**< 0.001**
Na (mg/d)	5724.88 ± 1653.15	6110.78 ± 1662.09	6239.57 ± 1566.09	6670.12 ± 1713.27	**0.06**
Dietary fiber (g/d)	49.88 ± 28.88	57.98 ± 22.65	58.55 ± 19.27	64.95 ± 28.94	**0.05**

DDS: Dietary Diversity Score, SSB: Sugar-sweetened beverages, SFA: Saturated fatty acids, PUFA: Polyunsaturated fatty acids, MUFA: Monounsaturated fatty acids, Ca: Calcium, Na: Sodium

The values are presented as mean ± SD.

Using one-way ANOVA.

Significant values are shown in bold.

**Table 5:** Risk for preeclampsia according to quartiles (Q) of the dietary quality index-international and dietary diversity score

	DQI-I					DDS			
	Q1	Q2	Q3	Q4	Ptrend	Q2	Q3	Q4	Ptrend
Model I	1 (Ref.)	0.62(0.25,1.51)	0.27(0.11,0.67)	0.09(0.03,0.24)	< 0.001	0.46(0.19,1.19)	0.61(0.26,1.44)	0.29(0.12,0.70)	0.049
Model II	1 (Ref.)	0.30(0.11,0.82)	0.12(0.04,0.34)	0.02(0.008,0.09)	< 0.001	0.29(0.11,0.75)	0.32(0.12,0.86)	0.10(0.03,0.33)	0.002
Model III	1 (Ref.)	0.31(0.10,0.92)	0.12(0.04,0.38)	0.02(0.005,0.08)	< 0.001	0.26(0.97,0.69)	0.30(0.11,0.84)	0.09(0.03,0.31)	0.001

DQI-I: Dietary Quality Index-International, DDS: Dietary Diversity Score

Model I: Crude, Model II: Adjusted for energy intake, Model III: Adjusted for age, education, energy intake, Pre-pregnancy BMI, family history

These values are odds ratio (95% CIs).

Obtained from logistic regression.

Significant values are shown in bold.

## Discussion

The study is the first to examine the association between DQI-I and DDS with the risk of preeclampsia. The results showed a significant inverse association between DQI-I and DDS with the risk of preeclampsia. This association remained significant after adjusting for potential confounders, including energy intake, socioeconomic characteristics, and pre-pregnancy BMI.

Extensive research indicates that assessing the overall quality of a diet, rather than focusing on specific foods or nutrients, is crucial for understanding its connection to diseases [30]. Various indicators of diet quality, such as DQI-I and DDS, have been employed to evaluate their association with non-communicable diseases like cardiovascular diseases (CVD) and hypertension [31, 32].

On the contrary, adopting a healthy dietary pattern has been linked to a decreased risk of preeclampsia. These findings suggest that promoting a healthy diet could potentially serve as a preventive measure for preeclampsia, reducing complications for both the mother and the baby in the future [33]. Additionally, a study demonstrated an inverse association between Iranian traditional dietary patterns and the occurrence of preeclampsia [34]. Similarly, adherence to a high-quality DASH-style diet showed a reverse relationship with the odds of preeclampsia in one study [35], and similar results were observed in another study with a Mediterranean-style diet [36].

While studies on the connection between DQI, DDS, and preeclampsia are limited, research has explored the link between dietary intake and cardio-metabolic diseases. Notably, a study indicated that a high dietary diversity may serve as a protective factor against cardio-metabolic disease risk factors within an urban cohort of South Asian adults [37].

In a separate study involving both US men and women [38], the association between diet quality scores and the risk of cardiovascular disease (CVD) was observed in both short-term and long-term contexts. The study revealed that the impact of reduced diet quality on CVD risk is more prominent during longer-term follow-up compared to short-term observations.

The study results indicate that a high intake of fruits and vegetables during pregnancy, attributed to micronutrients like antioxidants, vitamin B12, and folate, constitutes a healthy dietary pattern. This pattern has the potential to reduce the risk of preeclampsia, a significant pregnancy risk factor [39]. In predicting preeclampsia, inadequacies in certain nutrients such as protein, calcium, magnesium, selenium, and vitamins A, C, and D play a role. Notably, insufficient vitamin D increases the risk of preeclampsia in the second trimester [40]. Hence, maintaining a high-quality and diverse diet can contribute to preventing preeclampsia by ensuring the intake of essential vitamins and minerals.

In a case-control study that was conducted to investigate the associations of macro and micronutrients and antioxidants intake with preeclampsia an inverse and significant relationship was observed between preeclampsia and the intake of fruits, fiber, vitamin C, B-carotene, and olive oil. In the present study a significant and direct relationship was observed between preeclampsia and high intake of fat, saturated fat, and sodium [41]. The results obtained from a meta-analysis study showed that adherence to a dietary pattern high in vegetables, fruit, fish, whole grains and low in meat, processed food and sugar-sweetened foods has a significant potential to reduce the risk of preeclampsia in women [42]. Therefore, adopting nutritious dietary patterns and maintaining a high-quality diet can play a role in preventing preeclampsia according to our study.

In addition to dietary components, studies have shown that GWG and pre-pregnancy BMI can have a significant effect on the development of preeclampsia. In our study, pre-pregnancy BMI was not different between the two groups, while during pregnancy BMI was significantly higher in the preeclampsia group compared to the healthy control group. Additionally, women with preeclampsia had a higher GWG compared to the healthy women. A retrospective cohort study conducted by Gong et al. showed that women with a pre-pregnancy BMI of 24 or higher had a significantly higher risk of developing preeclampsia compared to women with a BMI below 24. Additionally, women who gained more weight during pregnancy than the recommended guidelines were also at an increased risk of developing preeclampsia [43]. Also, a population-based cohort survey of 98,820 women with singleton pregnancies in Slovenia revealed that excessive GWG was linked to higher odds of preeclampsia across all pre-pregnancy BMI categories, with a notable emphasis on underweight women [44]. These findings highlight the importance of maintaining a healthy weight before and during pregnancy to reduce the risk of preeclampsia.

This study has several strengths; it was the first to report the association between DQI-I and the risk of preeclampsia. We controlled for a wide range of confounding factors in the present study to establish an independent association between dietary scores and the risk of preeclampsia. In addition, the assessment of dietary quality by the DQI-I is simple and accurate compared to other methods because it adjusts for the effects of energy intake.

This study had some limitations. Despite using a validated FFQ, measurement errors were inevitable. Our pre-pregnancy weight data were self-reported. Another limitation is the case–control design of the study, which is subject to several biases, including selection and recall bias. Recall bias, in which subjects may recall their past diet in the context of preeclampsia diagnosis differently, is problematic because dietary assessment is performed after diagnosis. Therefore, the case-control design is the main limitation due to the inability to support cause-and-effect relationships.

## Conclusions

Based on our findings, there is a significant relationship between DDS, DQI-I, and the preeclampsia disease, and these patients have lower DDS and DQI-I compared to healthy individuals. In conclusion, increasing the quality and diversity of the diet may have potential beneficial effects in reducing the likelihood of developing preeclampsia. However, to achieve more accurate results, more laboratory investigations taking into account individual characteristics and more confounding variables in a larger population of these patients, or longer and prospective studies in this field, are needed.

## Data Availability

The datasets used and/or analyzed during the current study are available from the corresponding author upon reasonable request.
